# Structural and biochemical insights into the thermostable esterase Ta0887 from *Thermoplasma acidophilum*

**DOI:** 10.1002/2211-5463.70327

**Published:** 2026-08-21

**Authors:** Alejandro Delgado‐Rey, Miriam Livier Llamas‐García, Gabriela M. Montero‐Morán, Leticia Santos, Samuel Lara‐González

**Affiliations:** ^1^ IPICYT, División de Biología Molecular, Instituto Potosino de Investigación Científica y Tecnológica A.C. San Luis Potosí México; ^2^ Facultad de Ciencias Químicas Universidad Autónoma de San Luis Potosí México

**Keywords:** cap domain, carboxyl esterase, crystal structure, *Thermoplasma acidophilum*, thermostable esterase, α/β‐hydrolase

## Abstract

Microbial esterases are versatile and stable enzymes with a wide range of biotechnological applications. However, few esterases have been characterized from archaea, an important source of extremophilic enzymes. In this study, we report the biochemical characterization and crystal structure of Ta0887, a novel esterase from the thermoacidophilic archaeon *Thermoplasma acidophilum*. The protein was successfully cloned, expressed, and purified in *Escherichia coli*. Light scattering assays revealed that Ta0887 is a monomer in solution. Activity assays using *p*‐nitrophenyl (*p*‐NP) esters confirmed its esterase activity, showing a substrate preference for *p*‐NP hexanoate (C6). Furthermore, the substitution of Ser95 with alanine completely abolished enzymatic activity, thereby confirming its essential role as the nucleophilic residue of the catalytic triad. The enzyme exhibited optimal activity at 65 °C and pH 8.0. Notably, Ta0887 displayed high thermal stability, retaining 66% residual activity after incubation at 80 °C for 2 h, consistent with its thermal denaturation midpoint temperature of 80.6 °C. The crystal structure of Ta0887, resolved at 1.93 Å, revealed an α/β‐hydrolase core domain consisting of an eight‐strand β‐sheet, surrounded by seven α‐helices, and a cap domain comprising four α‐helices. Ta0887 features a large substrate‐binding pocket at the interface between the two domains that contains the conserved residues Ser95, Asp187, and His215 of the catalytic triad. Further analysis indicates that an efficiently packed hydrophobic core is a key feature for the observed thermostability. The findings from this study provide a basis for the future engineering of Ta0887 with the aim of enhancing its potential for industrial and biotechnological applications.

## Graphical abstract

In this study, a novel esterase from the thermoacidophilic archaeon *Thermoplasma acidophilum* was biochemically and structurally characterized. Our results demonstrate that Ta0887 is a highly thermostable esterase that preferentially hydrolyzes *p*‐nitrophenyl hexanoate and possesses an α‐helical cap domain that likely contributes to its substrate specificity. Ta0887 represents an attractive candidate for future enzyme engineering and biotechnological applications. The thermometer icon was obtained from SVG Repo under the CC0 license (https://www.svgrepo.com/svg/192961/thermometer‐temperature).

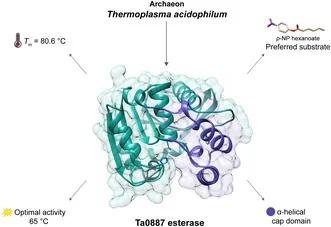

**Abbreviations**
Ade1: esterase from amazonian dark earth soil
AF‐Est2: esterase‐2 from *Archaeoglobus fulgidus*
AsFAE: feruloyl esterase from *Alistipes shahii*
ASU: asymmetric unit
CV: column volumes
DLS: dynamic light scattering
DSF: differential scanning fluorimetry
*E. coli*
: *Escherichia coli*
EaEst: esterase from *Exiguobacterium antarcticum*
ESTHER: ESTerases and α/β‐Hydrolases Enzymes and Relatives
HRV‐3C: human rhinovirus protease 3C
IDT: Integrated DNA Technologies
IPTG: isopropyl‐β‐D‐1‐thiogalactopyranoside
kDa: kilodaltons
LB: Luria–Bertani medium
ORF: open reading frame
PDB: Protein Data Bank
*p*‐NP: *p‐nitrophenyl*
RMSD: root‐mean‐square deviation
SD: standard deviation
SDS/PAGE: denaturing polyacrylamide gel electrophoresis
*T*_m_
: thermal denaturation midpoint
TtEst: esterase from *Thermogutta terrifontis*
TtEST2: esterase‐2 from *Thermogutta terrifontis*

Esterases, also known as carboxylesterases (EC3.1.1.1), are a class of enzymes found in all domains of life. Their primary function is to catalyze the hydrolysis of ester bonds, resulting in the formation of an alcohol and a carboxylic acid [1, 2, 3]. Both esterases (EC3.1.1.1) and lipases (triacylglycerol acylhydrolases, EC3.1.1.3), collectively known as lipolytic enzymes, exhibit the canonical α/β‐hydrolase fold. These enzymes contain a highly conserved catalytic triad, composed of a nucleophilic serine that is generally embedded within a G‐X‐S‐X‐G pentapeptide motif (where X can be any amino acid), as well as an aspartate or glutamate and a histidine [2, 4, 5]. However, esterases differ from lipases in that they show a preference for short‐chain acylglycerides (< 10 carbon atoms) and other simple esters [1, 6, 7, 8]. Furthermore, these enzymes can catalyze synthetic reactions in the absence of water or in the presence of organic solvents, such as esterification and transesterification [2, 9]. This versatility in catalysis, their high activity and stability in both aqueous and non‐aqueous systems, and their ability to act on various substrates without requiring cofactors explain the wide range of biotechnological applications for esterases [10, 11, 12]. In 2021, global demand for hydrolases was valued at $5.53 billion, with an annual growth projection of 6.6% from 2022 to 2030. Esterases led the hydrolase enzyme market with 34.3% of revenue in that year. This dominance can be attributed to their widespread use in industries such as food, pharmaceuticals, cosmetics, paper, and environmental management [8, 11, 13, 14].

In recent decades, significant efforts have been made to discover and characterize thermostable esterases derived from thermophilic microorganisms due to their ability to remain active in extreme conditions [4, 15, 16]. In this regard, most of the lipolytic enzymes studied and characterized have been derived from bacteria, with only a few originating from archaea. The natural habitats of archaea are extreme environments, including high (> 70 °C) or low temperatures (< 0 °C), highly alkaline (pH > 10) or acidic (pH < 5) conditions, and environments with extreme salinities (> 10% NaCl concentration). Consequently, archaea are regarded as a significant and promising source of extremophilic lipolytic enzymes [17]. Thermostable esterases have been characterized from the archaea *Picrophilus torridus* [18], *Aeropyrum pernix* [19, 20], *Nitrososphaera gargensis* [21], *Sulfolobus shibatae* [22], *Sulfolobus tokodaii* [23, 24], *Sulfolobus solfataricus* [25, 26, 27, 28], *Sulfolobus acidocaldarius* [29, 30], *Pyrobaculum* sp. 1860 [31], *Pyrobaculum calidifontis* [32], and *Archaeoglobus fulgidus* [4, 15, 17, 33, 34, 35].

The thermoacidophilic archaeon *Thermoplasma acidophilum* is a valuable source of thermostable enzymes with biotechnological applications. This microorganism has been key in elucidating three‐dimensional protein structures and molecular mechanisms due to its simplicity and molecular stability across a wide range of temperatures and pH values [36, 37, 38, 39, 40, 41, 42, 43]. Its optimal growth conditions are at a temperature of 59 °C and a pH between 1 and 2 [44]. The cells of this species are distinguished by a cytoplasmic membrane composed of a monolayer of tetraether lipids, which confers greater resistance to extremophilic conditions [45]. In addition, the chromosome of this microorganism has been fully sequenced, consisting of 1.56 million base pairs and 1509 open reading frames (ORFs). Approximately 55% of the ORFs in this microorganism encode proteins with either known or inferred functions [46], such as ORF Ta0887, which is predicted to be a protein related to triacylglycerol lipase (UniProt Primary Accession: Q9HJS7).

In this study, we performed the biochemical and structural characterization of the recombinant Ta0887 enzyme. Although Ta0887 is annotated as a putative lipase, our results demonstrate that it is a novel, highly thermostable esterase with an α‐helical cap domain that determines its substrate specificity. To our knowledge, this is the first esterase characterized from *T. acidophilum*. Our findings establish a solid foundation for future engineering of Ta0887, with a focus on enhancing its catalytic performance and expanding its applications in biotechnological processes.

## Materials and methods

### Bioinformatic analysis

The nucleotide sequence of Ta0887 was obtained from the NCBI (National Center for Biotechnology Information) database. The following bioinformatics tools were used to predict various physicochemical parameters of the protein *in silico*. The molecular weight, theoretical isoelectric point, and instability index were estimated using the protparam web tool (http://web.expasy.org/protparam/) [47]. The propensity to crystallize was evaluated using the CRYSTALP2 server (http://biomine.cs.vcu.edu/servers/CRYSTALP2/) [48]. The catalytic residues were identified through sequence alignment using the EMBOSS Needle tool on the EMBL‐EBI web server (https://www.ebi.ac.uk/Tools/psa/emboss_needle/) [49]. Secondary structure prediction was performed using the Jpred4 web server (https://www.compbio.dundee.ac.uk/jpred/) [50].

### Phylogenetic and sequence conservation analyses

A total of 52 representative protein sequences belonging to the 35 lipolytic enzyme families previously classified by Hitch and Clavel were retrieved from the UniProt Knowledgebase. Multiple sequence alignment was performed in mega version 12 [51] using the MUSCLE algorithm with default parameters. Pairwise sequence identities were calculated using clustal omega [52]. Phylogenetic relationships were inferred in MEGA 12 using the Maximum Likelihood method with the Le and Gascuel model of amino acid substitutions. The tree with the highest log‐likelihood score (−48 643.44) was selected. The initial tree for the heuristic search was generated by selecting the topology with the higher log‐likelihood between a Neighbor‐Joining tree and a maximum parsimony tree. The neighbor‐joining tree was constructed from a matrix of pairwise distances calculated using the p‐distance method, whereas the maximum parsimony tree corresponded to the shortest tree obtained from 10 independent searches initiated with randomly generated starting trees. The final dataset comprised 52 amino acid sequences and 865 aligned positions. Evolutionary analyses were performed in MEGA 12 using up to seven parallel computing threads. Pairwise sequence identity values were calculated independently using clustal omega. For evolutionary conservation analysis, a second multiple sequence alignment was generated using MAFFT with default parameters. The resulting alignment was uploaded to the ConSurf server [53], where residue‐specific evolutionary conservation scores were calculated using the Bayesian inference method. The information on the 52 sequences used in the analysis is provided in Table S1.

### Cloning, expression, and purification

The gene encoding the Ta0887 protein from *T. acidophilum* was optimized according to the codon usage of the *Escherichia coli* (*E. coli*) heterologous expression system. The sequence of the wild‐type gene (GenBank AL445065.1) and the optimized gene, with 12–15 bases added to each end to include the necessary cloning sites, are shown in Table S2. The gene was obtained as a double‐stranded DNA fragment (gBlock) from Integrated DNA Technologies (IDT) (https://www.idtdna.com/). It was cloned between the *Nde*I and *Hind*III restriction sites of a modified pET28 vector, in which the thrombin site was replaced with that of the human rhinovirus protease 3C (HRV‐3C). The ligation reaction was carried out using T4 ligase (Roche) at a 3 : 1 molar ratio. *E. coli* TOP10 cells were transformed with 2 μL of the ligation reaction and subsequently cultured on Luria‐Bertani (LB) solid medium supplemented with kanamycin (50 μg mL^−1^) at a temperature of 37 °C for a period of 16 h. The construct that contained the correct insert (pET28‐Ta0887) was identified by colony PCR and restriction enzyme assays and was subsequently confirmed by Sanger sequencing using T7 external oligonucleotides.

Protein expression and purification were performed as previously described, with modifications [54]. Protein expression was carried out in *E. coli* BL21 Star (DE3) cells with 0.4 mm isopropyl‐β‐d‐1‐thiogalactopyranoside (IPTG) for 4 h at 37 °C in LB medium supplemented with 50 μg mL^−1^ kanamycin. These conditions were selected based on preliminary expression optimization assays in which different IPTG concentrations, induction temperatures, and induction times were evaluated. The cells were harvested by centrifugation at 11 000 ***g*** for 10 min at 4 °C. The cell pellet was resuspended in 15 mL of lysis buffer (50 mm Tris/HCl, pH 8.0; 300 mm NaCl; 10 mm imidazole; 2 mm β‐mercaptoethanol) and disrupted by sonication on ice using 30 sonication cycles (15 s ON, 45 s OFF) at 50% amplitude. The soluble fraction was recovered by centrifugation at 15 000 ***g***  for 10 min at 4 °C. A two‐step chromatography procedure was used to perform the purification. First, the soluble fraction was loaded onto a pre‐equilibrated Ni‐NTA agarose column (Jena Bioscience, Thuringia, Germany) in lysis buffer. The column was then washed with 10 column volumes (CV), followed by elution in a single step with four CV of lysis buffer supplemented with 500 mm imidazole [54]. The protein‐containing fractions were collected and digested for 48 h at 4 °C with five units of HRV‐3C protease per milliliter of sample. For the second purification step, the protein was loaded onto a HiTrap SP column (GE Healthcare Life Sciences) in buffer A (20 mm HEPES, pH 7.0; 50 mm NaCl; 1 mm EDTA; 2 mm β‐mercaptoethanol) at a flow rate of 2 mL·min^−1^ using an ÄKTA Purifier system (Cytiva, Wilmington, DE, USA). Elution was performed using a linear gradient from 0% to 50% buffer B (20 mm HEPES, pH 7.0; 1 m NaCl; 1 mm EDTA; 2 mm β‐mercaptoethanol) over 20 CV. After the ion‐exchange chromatography step, fractions containing purified Ta0887 were pooled and stored at 4 °C for subsequent experiments. Protein expression and purity during the purification process were analyzed by denaturing polyacrylamide gel electrophoresis (SDS/PAGE) [55]. The Ta0887 Ser95‐Ala mutant, obtained as a gBlock from IDT, was cloned, expressed, and purified using the same procedure described for the wild‐type enzyme (Table S2).

### Dynamic light scattering

Dynamic light scattering (DLS) measurements were performed using a Zetasizer APS2000 instrument (Malvern Panalytical Ltd, Malvern, UK) with a final reaction volume of 50 μL. The protein was concentrated to 1 mg mL^−1^ by ultrafiltration using a Vivaspin Turbo 15 device with a 10‐kDa membrane (Sartorius AG, Göttingen, Germany) in purification buffer (buffer P; 20 mm HEPES, pH 7.0; 155 mm NaCl; 1 mm EDTA; 2 mm β‐mercaptoethanol). Prior to measurement, as suggested, the sample was centrifuged at 10 000 rpm for 4.5 h at 4 °C [56]. Fifteen 40‐s measurements were taken at 25 °C with a 5‐min preincubation period; the experiment was performed in triplicate. The molecular weight was estimated from the hydrodynamic particle radius using malvern zetasizer software, version 7.03 (Malvern Panalytical Ltd).

### Biochemical characterization

Enzyme activity assays were performed spectrophotometrically using *p*‐nitrophenyl esters (*p*‐NP) of various chain lengths, including *p*‐NP acetate (C2), *p*‐NP butyrate (C4), *p*‐NP hexanoate (C6), *p*‐NP octanoate (C8), *p*‐NP decanoate (C10), *p*‐NP laurate (C12), *p*‐NP myristate (C14), and *p*‐NP palmitate (C16). Stock solutions of each substrate at 10 mm were prepared in isopropanol and sonicated at 50% amplitude with 15‐s ON and 15‐s OFF pulses for 6 min at room temperature [4, 18, 57, 58]. Before use, the substrate stock solutions were equilibrated to room temperature and vortexed until clear, with no visible precipitate remaining. Unless otherwise indicated, the standard reaction mixture (1 mL) consisted of 50 mm HEPES buffer (pH 7.0), 0.1% gum arabic, 0.4% Triton X‐100, 0.25% polyvinyl alcohol, 0.4 mm substrate, and 1.5 μg mL^−1^ enzyme. The reaction was carried out at 40 °C for 4 min following a 5‐min preincubation at the same temperature before adding the enzyme. The reaction was stopped by adding 100 μL of 20% SDS. The amount of *p*‐NP released was determined by measuring the absorbance at 400 nm using a PerkinElmer Lambda XLS UV/VIS spectrophotometer [8, 59, 60]. An enzyme‐free control was included to correct for substrate autohydrolysis in all measurements. Enzyme activity was calculated using a calibration curve for different *p*‐NP concentrations prepared under the assay conditions. Each assay was performed in triplicate. One unit of enzyme activity (U) was defined as the amount of enzyme required to release 1 μmol of *p*‐NP per minute [61, 62].

To determine the optimal reaction temperature, an activity assay was conducted using *p*‐nitrophenyl butyrate at temperatures ranging from 25 to 90 °C. The optimal pH was determined by performing the activity assay with a three‐component buffer (0.05 m acetic acid, 0.05 m MES, and 0.1 m Tris), which kept the ionic strength constant over a pH range of 4.5–9.0 [63]. *p*‐NP exhibits two absorption maxima: one at approximately 320 nm, which is observed at pH values below 7.0 due to the relative abundance of its protonated form, and another at approximately 400 nm, which is observed at pH values of 7.0 or higher due to the predominance of its deprotonated form [64]. Thus, for pH values from 4.5 to 6.5, the amount of *p*‐NP released was determined by measuring the absorbance at 320 nm, while for pH values from 7.0 to 9.0, it was determined by measuring the absorbance at 400 nm. For this assay, *p*‐NP calibration curves at pH 4.5 and 5.5, measured at 320 nm, were used along with a curve at pH 7.5 whose absorbance was recorded at 400 nm.

The thermal stability of Ta0887 was evaluated by incubating the enzyme at temperatures ranging from 50 to 90 °C for 2 h, followed by incubation at 4 °C for 5 min, and then measuring the residual activity using *p*‐NP butyrate. To determine the thermal denaturation midpoint (*T*_m_), a differential scanning fluorimetry (DSF) assay was performed in an RT‐PCR 7500 Fast Kit thermocycler (Applied Biosystems, California, USA). Samples were prepared at a protein concentration of 0.1 mg mL^−1^ in buffer P with 5X SYPRO ORANGE and a final volume of 40 μL. The fluorescence signal was measured over a temperature range of 25–95 °C, in 1 °C increments. Fluorescence intensities of the protein‐containing samples were corrected by subtracting those of the protein‐free samples. The *T*_m_ was then determined by fitting the corrected fluorescence intensities to the Boltzmann equation. The experiments were performed in triplicate.

Enzyme kinetic assays were performed at pH 8.0 using *p*‐NP acetate at concentrations ranging from 0.5 to 10.0 mm and *p*‐NP butyrate, *p*‐NP hexanoate, and *p*‐NP octanoate at concentrations ranging from 0.1 to 5.0 mm. The corresponding *p*‐NP calibration curve was used, and the initial velocities were calculated by estimating the slope of the linear portion of the absorbance versus time data. The kinetic parameters *K*_m_ and *V*_max_ were calculated by nonlinear fitting of the data to the Michaelis–Menten equation using graphpad prism software (version 10.4.0, GraphPad Software, Boston, MA, USA).

### Crystallization, data collection, and structure determination

Crystallization screening was performed using the sitting‐drop vapor diffusion technique by mixing equal volumes of Ta0887 (8 mg mL^−1^) and the precipitants from the Crystal Screen I and II sparse matrix (Hampton Research, Aliso Viejo, CA, USA). The 96‐well plates were incubated at 14 °C and monitored using a stereomicroscope. Crystals suitable for X‐ray diffraction analysis were obtained after 24 h in 0.1 m HEPES‐Na (pH 7.5), 10% isopropanol, and 20% polyethylene glycol 4000. Crystals were cryoprotected through soaking in the crystallization solution augmented with 20% glycerol. A dataset from a single crystal was collected at 1.93 Å resolution using a MicroMax 007HF rotating anode generator (Cu Kα, l = 1.5418) equipped with a DECTRIS‐PILATUS 3 R/200K‐A detector at 100 K.

The collected data were indexed, integrated, and scaled using the HKL3000 software suite [65]. The crystal was trigonal and belonged to the space group P3_2_21. Structure factor data were analyzed using the *phenix.xtriage* program from the Phenix software suite [66]. The structure of Ta0887 was solved by molecular replacement using phaser [67], with the structure of the ht‐CarCJ3 hydrolase (PDB: 1J1I) used as the search model. The final coordinates were obtained by iterating between refinement with PHENIX and manual model building to improve the fit to likelihood‐weighted electron density maps using coot [68]. Water molecules were added where supported by both chemistry and geometry using difference |*F*_obs_| − |*F*_calc_| electron density > 5σ. The final model was validated with MolProbity, available at http://molprobity.biochem.duke.edu/ [69]. Electrostatic surface potentials were calculated at pH 8.0 using the Adaptive Poisson‐Boltzmann Solver software suite [70]. The visualization of electrostatic and hydrophobic surfaces was conducted in UCSF Chimera [71]. Cavity analysis was performed using the generate‐mode of PDBsum [72] or CASTpFold server with the default probe radius of 1.4 Å (https://cfold.bme.uic.edu/castpfold/) [73], and the resulting output files were visualized using pymol [74]. To determine the number of hydrogen networks, salt bridges, and hydrophobic clusters, PDB files without water and ligands were used; the data were obtained from the proteintools server (https://proteintools.uni‐bayreuth.de/) [75]. PDB accession number, data collection, and final model statistics are listed in Table 3.

## Results

### Bioinformatic analysis of Ta0887

The genome of *Thermoplasma acidophilum* was described by A. Ruepp and colleagues in 2000 [46]. A total of 1509 open reading frames were reported, including several α/β‐hydrolases of unknown specificity; only Ta0887 was annotated as a putative triacylglycerol lipase‐related protein. A search for α/β‐hydrolases in the genome of *T. acidophilum* using the ESTHER database (ESTerases and α/β‐Hydrolases Enzymes and Relatives) allowed for the identification of eight proteins. Of these, two have already been characterized and correspond to an F1 proline iminopeptidase [76] and a CIB‐like hydrolase whose structure has already been determined (Ta0194, PDB: 3BDI). The remaining six proteins have not yet undergone experimental characterization. These include a putative dienelactone hydrolase (Ta0228); two possible feruloyl esterases (Ta1047, Ta1402); two proteins for which no description is available (Ta0664, Ta1481); and Ta0887, which is predicted to be a triacylglycerol lipase‐related protein. The *Ta0887* gene was selected for further analysis with the aim of determining its phylogenetics and structural characteristics.

**Fig. 1:**
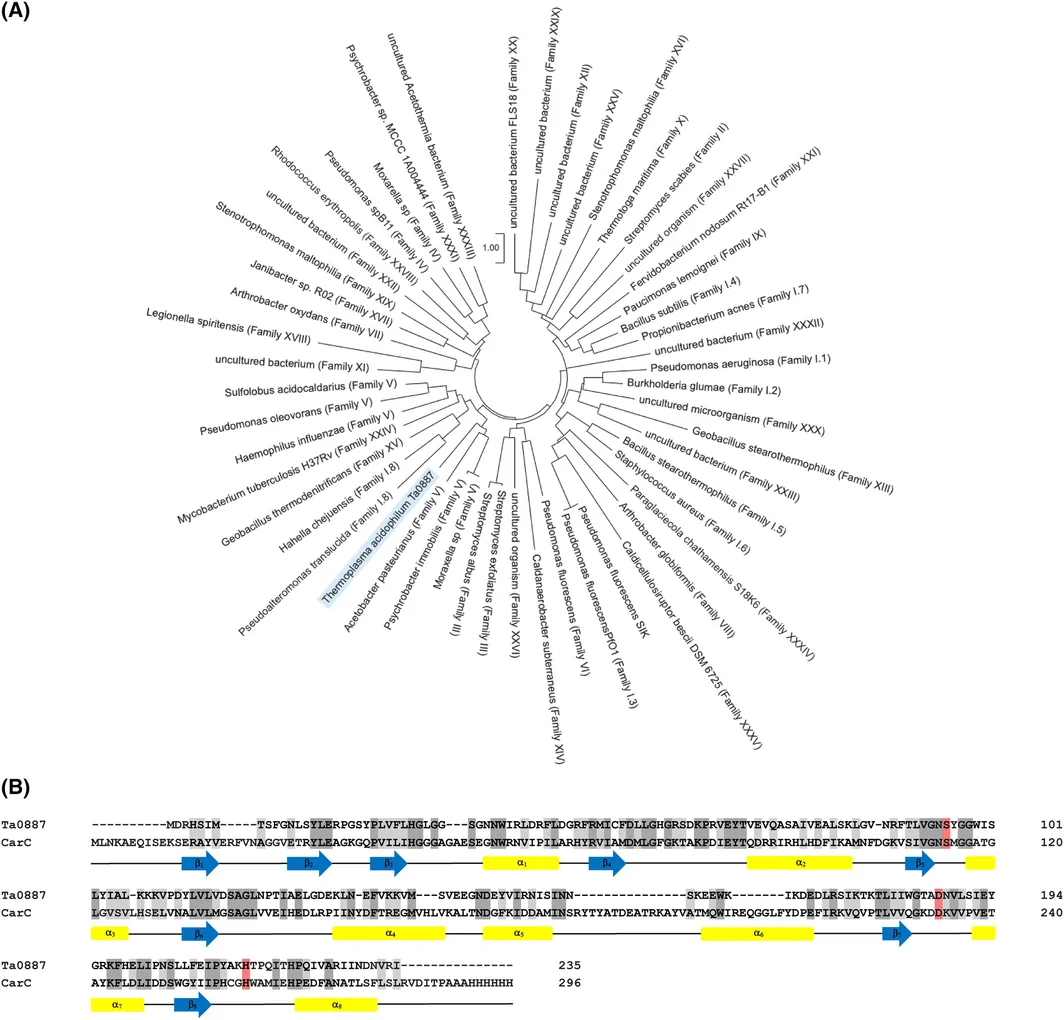
Phylogenetic analysis and sequence alignment of Ta0887. (A) Phylogenetic analysis of Ta0887 was performed using the maximum likelihood method. Fifty‐two sequences representing the 35 families of bacterial lipolytic enzymes (I–XXXV) described by Hitch and Clavel [5, 78] were used. The Ta0887 protein from *T. acidophilum* is highlighted in blue and is grouped within Family V. The phylogenetic analysis was performed using MEGA 12 [51]. (B) Sequence alignment of the Ta0887 protein with the ht‐CarCJ3 hydrolase from *Janthinobacterium*. The catalytic residues of CarC are conserved at the corresponding positions Ser95, Asp187, and His215 in Ta0887 (shaded in red) [79]. Identical residues are shaded in dark gray, whereas similar residues are shaded in light gray. The sequence alignment was performed using the EMBOSS Needle tool on the EMBL‐EBI web server (https://www.ebi.ac.uk/jdispatcher/psa/emboss_needle) [49]. The secondary structure prediction of Ta0887, performed using the Jpred4 web server (https://www.compbio.dundee.ac.uk/jpred/), is shown below the alignment [50]. The yellow rectangles indicate α‐helices, while the blue arrows represent β‐strands.

In 1999, Arpigny and Jaeger classified bacterial lipolytic enzymes into eight families based on the similarity of amino acid sequences and physiological properties (Families I–VIII) [6, 77]. This classification has recently been expanded to include 35 families of lipolytic enzymes (Families I–XXXV) [5, 78]. Phylogenetic analysis, performed using the 35‐family classification, indicates that Ta0887 belongs to Family V. The closest related sequence identified in this analysis corresponds to the Est2 esterase from *Acetobacter pasteurianus* (Fig. 1A).

Family V comprises lipolytic enzymes from mesophilic (*Haemophilus influenzae*, *Pseudomonas oleovorans*, and *Acetobacter pasteurianus*), psychrophilic (*Moraxella* sp. and *Psychrobacter immobilis*), and thermophilic (*Sulfolobus acidocaldarius*) microorganisms [6]. Ta0887 exhibits 39.4% amino acid sequence similarity with the putative esterase from *H. influenzae* (GenBank AAC21862), 37.3% with the lipase from *Moraxella* sp. (GenBank CAA37863), and 35.9% with the lipolytic enzyme from *S. acidocaldarius* (GenBank AAC67392). Furthermore, enzymes belonging to this family exhibit a significant similarity in amino acid sequence (20–25%) with several non‐lipolytic bacterial enzymes, including epoxide hydrolases, dehalogenases, and haloperoxidases [6]. This was observed in Ta0887, which exhibits ~ 30% sequence similarity with multiple epoxide hydrolases (PDB: 7AC0), dehalogenases (PDBs: 3WI7, 5MXP, 1Y37), and haloperoxidases (PDBs: 3FOB, 1A7U, 1BRO). Taken together, the classification of Ta0887 within Family V and the identification of the Est2 esterase from *A. pasteurianus* and a putative esterase from *H. influenzae* as its closest homologs suggest that Ta0887 is an esterase.

According to the GenBank database, the ORF *Ta0887* consists of 708 base pairs encoding a 235‐amino acid protein. The molecular weight of the protein, estimated using protparam, was 26.7 kDa. Its theoretical isoelectric point was 7.03, and its instability index (II) was 20.3, which allows it to be considered stable [47]. The propensity to crystallize, estimated using the CRYSTALP2 web server, was favorable with a confidence score of 0.59 [48]. In addition, a search for structural homologs was performed using the mmseqs2 method in the Protein Data Bank (PDB). This analysis identified the ht‐CarCJ3 hydrolase from *Janthinobacterium* sp. strain J3 (PDB: 1J1I) as the closest protein with a known experimental structure, with 41.8% similarity [79]. The alignment of Ta0887 sequence with that of ht‐CarCJ3 hydrolase allowed for the identification of residues Ser95, Asp187, and His215 as the catalytic triad, with Ser95 located in the conserved G‐X‐S‐X‐G motif, which is characteristic of lipolytic enzymes (Fig. 1B). As expected, these residues are highly evolutionarily conserved among members of Family V (Fig. S1).

### Cloning, expression, and purification of the recombinant Ta0887 protein

The *Ta0887* gene sequence was optimized for expression in *E. coli* and cloned into the pET28 expression vector. The resulting pET28‐Ta0887 construct was confirmed by sequencing. Expression assays were performed in *E. coli* BL21 Star (DE3) cells at different temperatures, induction times, and IPTG concentrations. Optimal expression conditions were obtained with 0.4 mm IPTG for 4 h at 37 °C (data not shown).

**Fig. 2:**
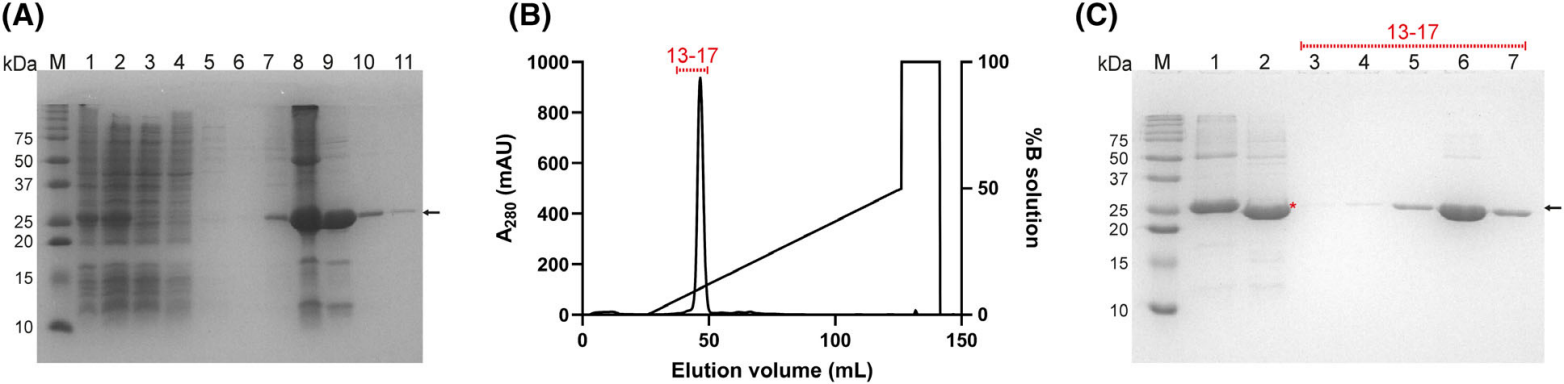
Purification of Ta0887 by two‐step liquid chromatography. (A) Denaturing polyacrylamide gel electrophoresis (SDS/PAGE) analysis of Ta0887 purified by nickel affinity chromatography. Samples were stained with Coomassie R250. M: molecular weight marker, 1: cell sonication fraction, 2: soluble protein fraction, 3: flow‐through fraction, 4–5: wash fractions, 6–11: proteins eluted with 500 mm imidazole. (B) Chromatogram of Ta0887 purification by ion‐exchange chromatography. The right *y*‐axis represents the percentage of buffer B, the left *y*‐axis represents the absorbance at 280 nm, and the *x*‐axis represents the elution volume. The fractions collected from the elution are marked in red. (C) SDS/PAGE analysis of the fractions corresponding to the peak shown in panel (B). M: molecular weight marker, 1: protein purified by nickel affinity, 2: protein treated with human rhinovirus protease 3C, 3–7: fractions corresponding to the peak in the chromatogram. The black arrow in (A), as well as the black arrow and red asterisk in (C), indicate the band corresponding to Ta0887.

The recombinant protein was successfully purified using a two‐step liquid chromatography procedure. In the first step, using nickel affinity chromatography, the protein eluted with 500 mm imidazole (Fig. 2A). The fractions containing the protein were subsequently pooled and treated with human rhinovirus protease 3C to remove the 6XHis tag present at the N‐terminus (Fig. 2C). In the second step, the protein was purified by ion‐exchange chromatography, resulting in the elution of Ta0887 at an average concentration of 155 mm NaCl (Fig. 2B,C). The amount of purified protein, after combining the protein‐containing fractions, was 9.6 milligrams per liter of culture.

### The oligomeric state of Ta0887 in solution

**Fig. 3:**
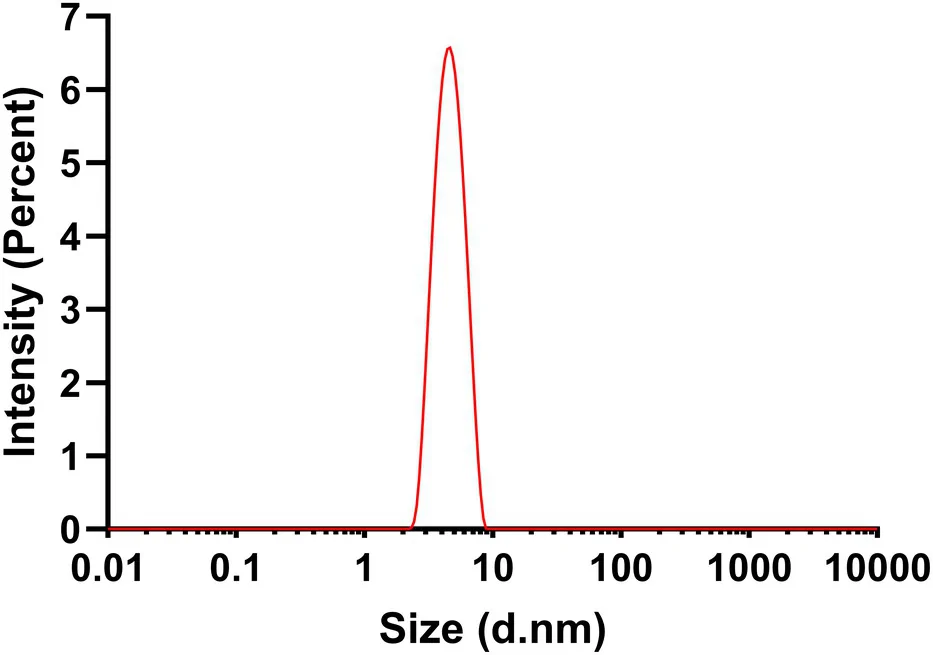
Determination of the oligomeric state of Ta0887 in solution by dynamic light scattering. The assay was performed at a protein concentration of 1 mg mL^−1^ in 20 mm HEPES (pH 7.0), 155 mm NaCl, 1 mm EDTA, and 2 mm β‐mercaptoethanol. The *y*‐axis corresponds to intensity, while the *x*‐axis corresponds to particle diameter. The analysis showed a single peak corresponding to a molecular weight of 25.4 ± 1.0 kDa, which is consistent with the theoretical molecular weight of Ta0887.

The estimated molecular weight of Ta0887 was 26.7 kilodaltons (kDa). During the purification process, an intense band around 25 kDa was observed by SDS/PAGE. However, this molecular weight was determined under denaturing conditions. To evaluate the molecular weight and oligomeric state of the protein under native conditions, dynamic light scattering experiments were performed. In these experiments, a hydrodynamic radius of 2.4 ± 0.6 nm was determined for Ta0887, corresponding to an estimated molecular weight of 25.4 ± 1.0 kDa (Fig. 3). Therefore, it was concluded that Ta0887 is a monomer under native conditions.

### Ta0887 is a thermostable esterase with preference for *p*‐NP hexanoate as substrate

**Fig. 4:**
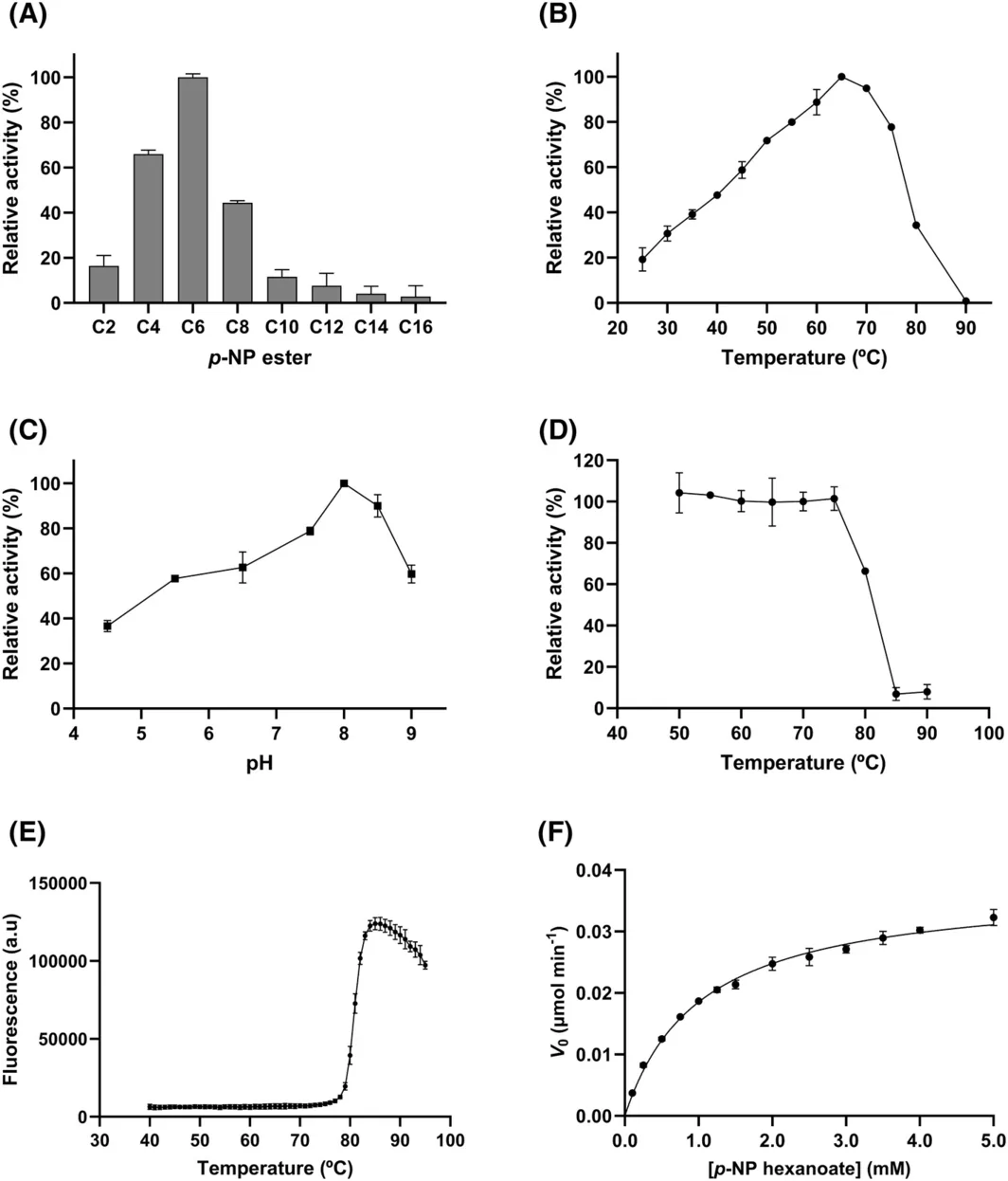
Biochemical properties of Ta0887. (A) The substrate specificity of Ta0887 was evaluated using different *p‐nitrophenyl* (*p*‐NP) esters (C2–C16). The activity of Ta0887 toward *p*‐NP hexanoate (C6) was set as 100%. (B) Effect of temperature on the activity of Ta0887. (C) Effect of pH on the activity of Ta0887. In both panels (B) and (C), the point at which the highest activity was observed was set as 100%. (D) Thermal stability of Ta0887. The enzyme was preincubated for 2 h at temperatures ranging from 50 to 90 °C, after which its residual activity was measured. The activity of Ta0887 without preincubation was considered 100%. (E) Thermal denaturation curve of Ta0887 in buffer P (20 mm HEPES, pH 7.0; 155 mm NaCl; 1 mm EDTA; 2 mm β‐mercaptoethanol). The estimated thermal denaturation midpoint (*T*_m_) value was calculated by fitting the data to the Boltzmann equation. The *y*‐axis corresponds to fluorescence in arbitrary units, and the *x*‐axis corresponds to temperature. (F) Initial velocities of Ta0887 as a function of *p*‐NP hexanoate (C6) concentration. The kinetic parameters *K*_m_ and *V*_max_ were determined through a non‐linear regression analysis with the Michaelis–Menten equation. In all panels, the results correspond to the average of three independent experiments, and the error bars represent the standard deviation.

To determine the substrate specificity of Ta0887, its activity was evaluated using *p*‐nitrophenyl (*p*‐NP) esters of varying chain lengths, ranging from *p*‐NP acetate (C2) to *p*‐NP palmitate (C16). Ta0887 displayed a strong preference for short‐ to medium‐chain substrates (C2–C8), exhibiting the highest specific activity with *p*‐NP hexanoate (C6; Table 1; Fig. 4A). High hydrolytic activity was also observed with *p*‐NP butyrate (C4), at 66% of the maximum activity. In contrast, low levels of activity were observed with *p*‐NP decanoate, *p*‐NP laurate, *p*‐NP myristate, and *p*‐NP palmitate (C10–C16; Table 1; Fig. 4A). These findings confirmed that Ta0887 is indeed an esterase.

**Table 1:** Substrate specificity of Ta0887 toward *p*‐NP esters of different chain lengths. Values represent mean ± standard deviation (SD) of three independent experiments. Relative activity was calculated considering the activity toward *p*‐NP hexanoate (C6) as 100%.

Substrate	Chemical structure	Specific activity (U·mg^−1^)	Relative activity (%)
*p*‐NP acetate (C2)	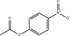	1.1 ± 0.3	16 ± 5
*p*‐NP butyrate (C4)	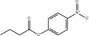	4.6 ± 0.1	66 ± 2
*p*‐NP hexanoate (C6)	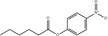	7.0 ± 0.1	100 ± 1
*p*‐NP octanoate (C8)	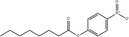	3.1 ± 0.1	44 ± 1
*p*‐NP decanoate (C10)	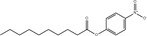	0.8 ± 0.2	12 ± 3
*p*‐NP laurate (C12)	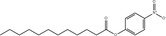	0.5 ± 0.4	8 ± 5
*p*‐NP myristate (C14)	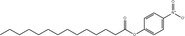	0.3 ± 0.2	4 ± 3
*p*‐NP palmitate (C16)	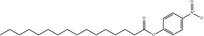	0.2 ± 0.3	3 ± 5

The optimal reaction temperature for Ta0887 was determined to be 65 °C, after evaluating its activity over a temperature range of 25–90 °C (Fig. 4B). At the optimal growth temperature of *T. acidophilum* (~ 60 °C), Ta0887 exhibited 89% of its maximum activity. The activity decreased to 34% at 80 °C and was practically negligible at 90 °C. The optimal reaction pH was determined to be 8.0 after measuring its activity over a pH range of 4.5–9.0 (Fig. 4C). The enzyme showed activity levels above 60% within a temperature range of 50–75 °C and a pH range of 6.5–8.5, indicating that Ta0887 exhibits a wide range of reaction temperatures and pH values.

To evaluate the thermal stability, Ta0887 was incubated for 2 h at temperatures ranging from 50 to 90 °C. It was then cooled to 4 °C before measuring its residual activity. No loss of activity was observed up to 75 °C. At 80 °C, Ta0887 showed 66% residual activity, and at higher temperatures, activity was virtually undetectable (Fig. 4D). These results were consistent with those obtained in a differential scanning fluorimetry assay. In this assay, the apparent midpoint of thermal denaturation (*T*_m_) of Ta0887, in the buffer in which it was purified, was 80.6 ± 0.2 °C (Fig. 4E). The protein exhibited a sharp transition curve between 72 and 85 °C, suggesting a homogeneous and compact folding. These results indicate that Ta0887 is thermostable and active up to 80 °C.

The kinetic parameters of Ta0887 were determined for *p*‐NP acetate, *p*‐NP butyrate, *p*‐NP hexanoate, and *p*‐NP octanoate at pH 8.0. The enzyme exhibited classical Michaelis–Menten kinetics, with *p*‐NP hexanoate showing the lowest *K*_m_ and the highest catalytic efficiency. For the other three substrates, the *K*_m_ values increased in the order *p*‐NP octanoate, *p*‐NP butyrate, and *p*‐NP acetate, whereas catalytic efficiency decreased in the order *p*‐NP butyrate, *p*‐NP octanoate, and *p*‐NP acetate. These findings further supported *p*‐NP hexanoate as the preferred substrate among those evaluated (Fig. 4F; Table 2).

**Table 2:** Kinetic parameters of Ta0887 toward various *p*‐NP esters. Kinetic parameters were determined at pH 8.0 under standard assay conditions by fitting the data to the Michaelis–Menten equation. Values represent mean ± SD of three independent experiments. Kinetic parameters for substrates from *p*‐NP decanoate to *p*‐NP palmitate could not be determined due to lack of substrate saturation or insufficient activity.

Substrate	*K*_m_ (mm)	*V*_max_ (μmol min^−1^)	*k*_cat_ (s^−1^)	*k*_cat_/*K*_m_ (mm ^−1^·s^−1^)
*p*‐NP acetate	16.1 ± 6.6	0.056 ± 0.020	16.6 ± 6.1	1.0 ± 0.1
*p*‐NP butyrate	2.5 ± 0.2	0.045 ± 0.002	13.4 ± 0.5	5.4 ± 0.3
*p*‐NP hexanoate	1.0 ± 0.1	0.037 ± 0.002	11.1 ± 0.5	10.9 ± 0.5
*p*‐NP octanoate	1.7 ± 0.2	0.017 ± 0.001	5.0 ± 0.4	3.0 ± 0.1

### The catalytic residue Ser95

**Fig. 5:**
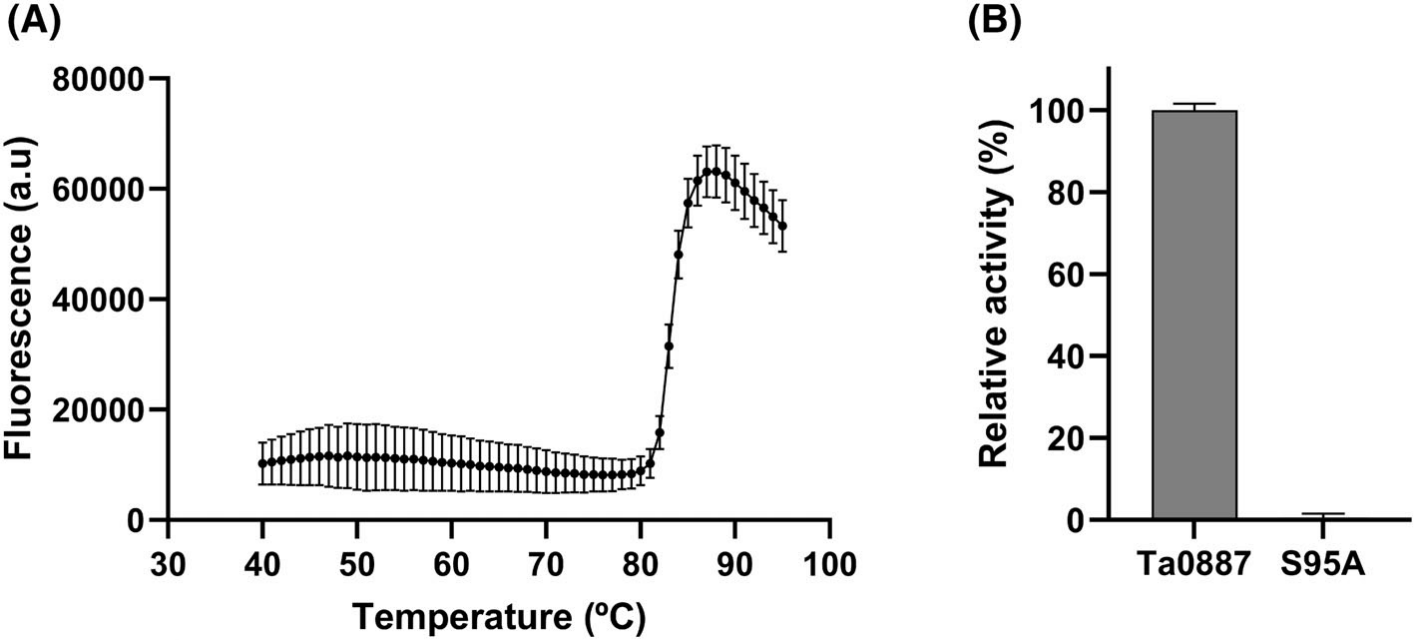
Thermal stability and relative activity of the Ser95‐Ala mutant. (A) Thermal denaturation curve of the Ser95‐Ala mutant in buffer P (20 mm HEPES, pH 7.0; 155 mm NaCl; 1 mm EDTA; 2 mm β‐mercaptoethanol). The estimated thermal denaturation midpoint (*T*_m_) value was calculated by fitting the data to the Boltzmann equation. (B) Relative activity of Ta0887 and the Ser95‐Ala mutant with *p*‐NP hexanoate. The activity of Ta0887 was set to 100%. The data represent the mean ± standard deviation of three independent replicates.

To validate the role of Serine at position 95, identified as the catalytic residue in the bioinformatic analysis, a point mutation to Alanine (Ser95‐Ala) was generated. This construct was expressed and purified using the same methodology described for the wild‐type enzyme (data not shown). The thermal stability of the Ser95‐Ala mutant, as determined by DSF, exhibited a value comparable to that observed for the wild‐type enzyme (*T*_m_ of 83.2 ± 0.1 °C; Fig. 5A). Furthermore, a single peak was observed by DLS, corresponding to an estimated molecular weight of 32.3 ± 3.0 kDa, consistent with the presence of the monomer in solution (data not shown). Subsequently, the activity of the Ser95‐Ala mutant was determined using *p*‐NP hexanoate as a substrate. As anticipated, the mutant exhibited no discernible activity under identical experimental conditions used for the wild‐type enzyme (Fig. 5B). These results suggest that the mutation at position 95 does not affect protein folding and confirm the central role of the nucleophilic serine in the catalytic activity of Ta0887.

### Overall structure of Ta0887

**Fig. 6:**
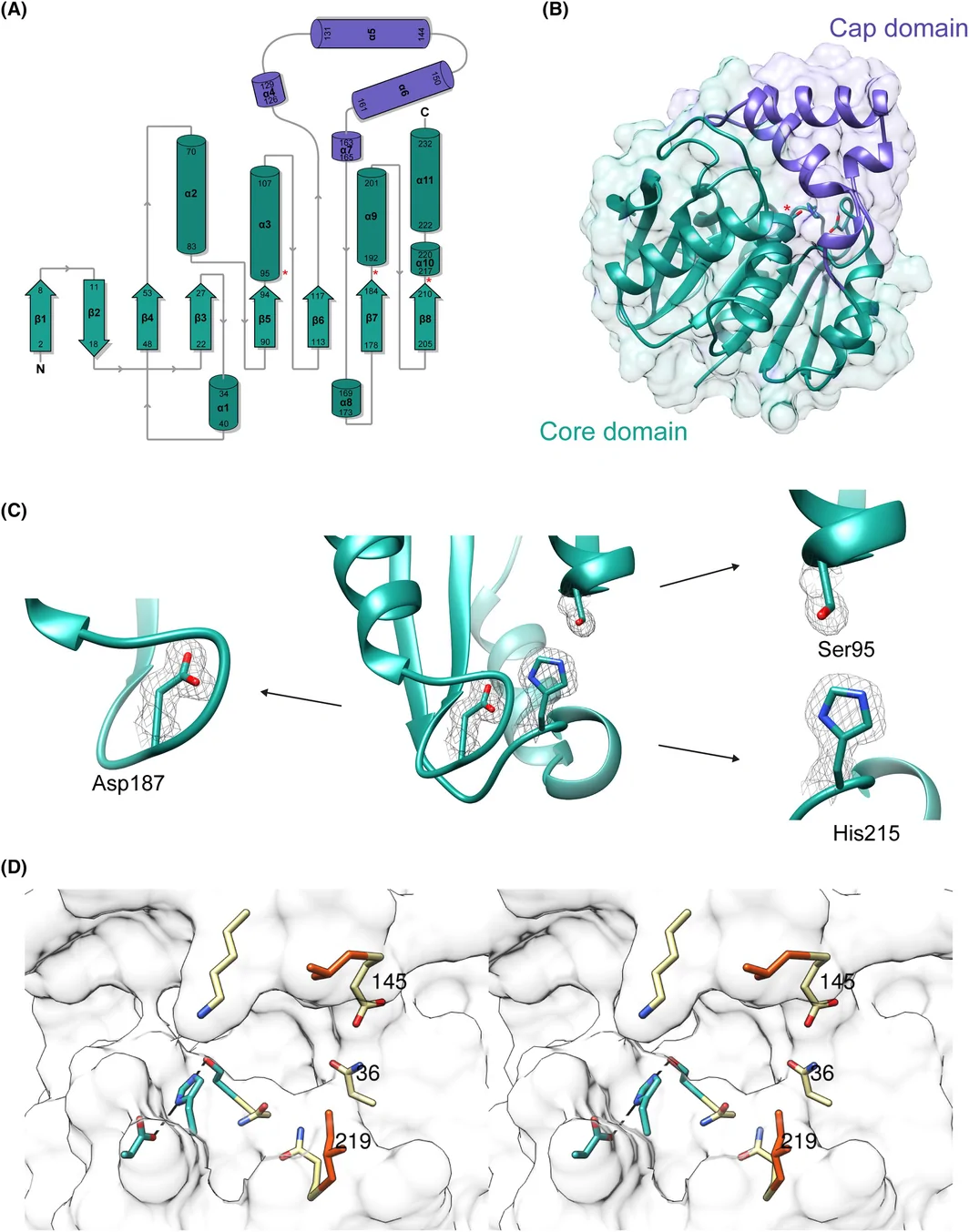
Crystal structure of Ta0887. The core and cap domains are shown in turquoise and violet, respectively. (A) The topology of Ta0887 consists of eight β‐strands (arrows) and 11 α‐helices (cylinders). The location of the catalytic residues is indicated by a red asterisk. (B) Ribbon representation of the crystal structure of Ta0887. The core domain, which has an α/β‐hydrolase fold, is shown oriented downward in turquoise; at the top, the α‐helical cap domain is shown in violet. The catalytic Ser95 residue is indicated by a red asterisk (C) A close‐up view of the active site is presented, with the conserved residues of the catalytic triad Ser95, His215, and Asp187 represented as sticks. The arrows indicate enlarged views of the residues, showing their corresponding 2*mF*_o_ *‐DF*_c_ electron density maps contoured at 1.5 σ. (D) Wall‐eye stereo view of the putative carboxyl‐binding pocket; key residue side chains are shown: the catalytic triad in turquoise, polar residues in yellow, and hydrophobic residues in orange. Ile239, Asn35, and Glu145 restrict the length of the pocket.

The crystal structure was determined using the molecular replacement method, based on the structure of the homolog ht‐CarCJ3 protein from *Janthinobacterium* sp. strain J3 (PDB: 1J1I). The final model was refined to a resolution of 1.93 Å with *R*_work_ and *R*_free_ values of 0.170 and 0.199, respectively (Table 3). The model geometry was evaluated using MolProbity [69], which indicated high stereochemical quality, with 96.8% of the residues in the favorable regions of the Ramachandran plot. The asymmetric unit (ASU) comprises two monomers, with a root‐mean‐square deviation (RMSD) of 0.34 Å for the Ca atoms. The main structural difference between the two monomers is observed in the region of residues 128–146, which form part of the cap domain. The Ta0887 structure comprises a core domain with the canonical α/β‐hydrolase fold (residues 1–123 and 168–235) and a cap domain located between residues 123 and 168 (residues 124–167). The core domain is composed of an eight‐strand β‐sheet (β1–β8), with β2 being antiparallel, surrounded by seven α‐helices forming an α/β/α sandwich. The α‐helices α1, α10, and α11 are located on one side of the sandwich, while the α‐helices α2, α3, α8, and α9 are located on the other. Meanwhile, the cap domain is formed by helices α4 to α7 (Fig. 6A,B).

**Table 3:** X‐ray diffraction data collection and refinement statistics.

	Ta0887
Data collection	
Space group	P3_2_21
Unit cell dimensions	
*a, b, c* (Å)	117.05, 117.05, 91.71
α, β, γ (°)	90, 90, 120
Wavelength	1.54178
Resolution (Å)	50.00–1.93 (1.96–1.93)^a^
Matthews coefficient (Å^3^ Da^−1^)	3.43
Total no. of reflections	624 532
No. of unique reflections	54 668
Completeness (%)	100 (100)^a^
*R*_merge_	0.120 (0.937)^a^
Mean *I*/σ(*I*)	18.9 (2.6)^a^
Average redundancy	11.4 (8.3)^a^
Refinement	
Resolution (Å)	50.00–1.93
No. of reflections	54 617
*R*_work_/*R*_free_	0.170/0.199^b^
Average B‐factor (Å^2^)	
Overall	19.39
From Wilson plot	18.05
No. of monomers in ASU	2
No. of residues	469
No. of atoms	7727
No. of water molecules	308
RMS deviations from ideal	
Bond lengths (Å)	0.012
Bond angles (°)	1.131
Clashscore	1.35^c^
Favored rotamers (%)	96.03^c^
Poor rotamers (%)	0.00^c^
Ramachandran plot statistics (%)	
Favored regions	96.8^c^
Outliers	0.00^c^
PDB accession code	10XN

^a^ The values in parentheses correspond to the last resolution shell

^b^ *R*_free_ was calculated using an independent subset of 5% of the reflections excluded during refinement

^c^ Analysis of steric clashes, favorable rotamer conformations, and the Ramachandran plot was performed using MolProbity.

### The substrate‐binding pocket and the catalytic site

The substrate‐binding pocket is a large cavity located at the interface between the cap domain and the α/β‐hydrolase domain (Fig. 6B,C). The cavity is lined by amino acids located on β5, α4, α5, α9, α10, the loop between β3 and α1, and the loop between β8 and α10. In contrast to other homologous esterases, such as AsFAE from *Alistipes shahii* (PDB: 7DQ9), whose cap domain covers the substrate‐binding pocket, in Ta0887 the α4 pushes the cap outward, resulting in an open substrate‐binding pocket exposed to the solvent. Furthermore, Ta0887 does not have a tunnel connecting the active site to the surface, as is observed in other esterases from Family V, such as EprEst (PDB: 7C4D) [80].

The substrate‐binding pocket contains the active site, which consists of residues Ser95, His215, and Asp187 that form the catalytic triad. Ser95 is part of the conserved G‐X‐S‐X‐G motif characteristic of esterases and is located in the nucleophilic elbow between β5 and α3. The other two residues of the catalytic triad are located in adjacent loops: His215 is located in the loop connecting β8 to α10, and Asp187 is situated in the loop between β7 and α9. The following interactions stabilize the residues of the catalytic triad. The Oγ atom of Ser95 interacts with the pyridine nitrogen (Nε2) of His215 via a hydrogen bond (2.7 Å). Meanwhile, the Nδ1 of His215 interacts with the Oδ2 of Asp187 via a salt bridge (2.9 Å).

If we take Ser95 as a reference point, behind this residue and toward the hydrolase core lies the oxyanion hole, which is formed by the main‐chain nitrogens of Tyr96 and Leu30. A structural comparison with the esterase TtEst2 from *Thermogutta terrifontis* (PDB: 5AOB) allowed us to identify the putative carboxyl‐binding pocket. This site, which extends from Ser95 toward the solvent, is lined by polar and charged residues such as Asn94, Gln218, Asn36, and Glu145, and by hydrophobic residues such as Leu30, Phe137, and Ile219 (Fig. 6D). As a result, the carboxyl‐binding pocket exhibits a positively charged surface that extends throughout the entire cavity (Fig. S2). The length of the carboxyl‐binding pocket is defined by residues Asn36, Glu145, and Ile219, which are located at the opposite end from where Ser95 is located. A comparison with TtEst2 indicates that the pocket can accommodate short‐chain esters, such as butyrate. Therefore, these residues play a key role in the specificity toward short‐chain substrates (Fig. 6D). Other conserved residues in the vicinity of Ser95 include Ala120, Leu190, and Tyr194. However, their function could not be established (Fig. S1).

A notable feature of the Ta0887 structure is the size of the substrate‐binding pocket. We therefore determined the volume of this region using PDBsum tools [72, 81] and compared the results with structural homologs identified using the DALI server [82]. The esterases selected for analysis included those from *Exiguobacterium antarcticum* (EaEst, PDB: 5H3H), *Thermogutta terrifontis* (TtEst, PDB: 4UHC), *Alistipes shahii* (AsFAE, PDB: 7DQ9), an esterase identified in a metagenomic library of Amazonian dark earth soils (Ade1, PDB: 8V16), and one from *Archaeoglobus fulgidus* (AF‐Est2, PDB: 5FRD). Cleft analysis with PDBsum revealed significant variations in cavity size, ranging from 966.1 to 5423.6 Å^3^. Despite having the fewest residues, Ta0887 exhibits a notably large cavity compared to the other esterases, with the exception of Ade1, which is larger. This finding suggests that Ta0887 may utilize other substrates, likely of larger volume or branched, that have yet to be identified (Table S3, Fig. S2).

### Ta0887 thermostability

To gain insights into the molecular mechanisms by which Ta0887 is thermostable, a variety of structural features were analyzed, including hydrogen bonds, salt bridges, hydrophobic clusters, and the size and number of buried cavities. The results were then compared with the mesophilic esterase AsFAE (PDB: 7DQ9) and the thermophilic esterase AF‐Est2 (PDB: 5FRD).

**Fig. 7:**
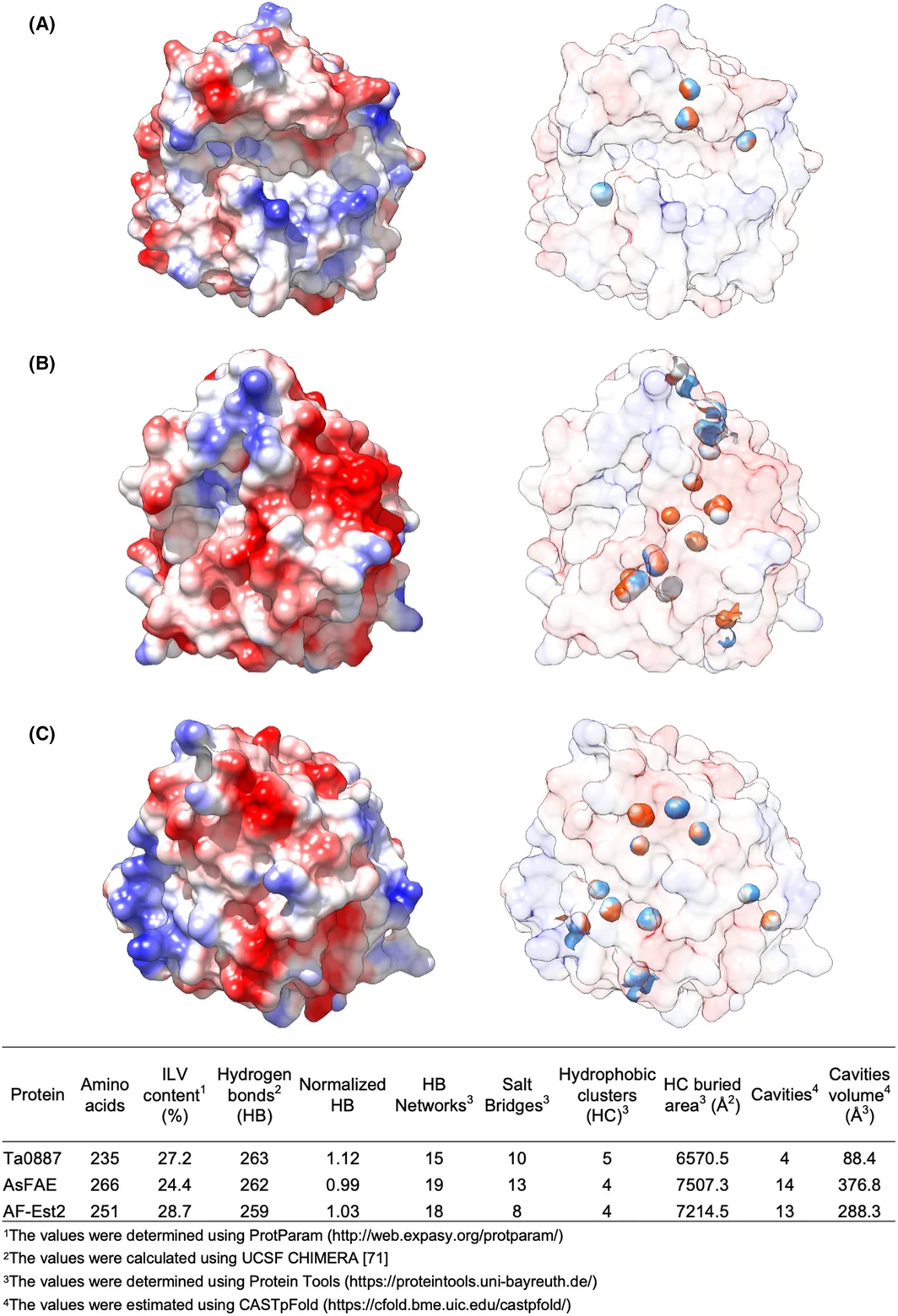
Structural properties involved in the thermostability of Ta0887. (A) The electrostatic surface representation of Ta0887 is shown on the left, while the transparent surface on the right allows for observation of the four cavities present in the protein's α/β‐hydrolase core. Similarly, (B) shows the 14 cavities present in the mesophilic esterase from *Alistipes shahii* (AsFAE), and (C) shows the 13 cavities observed in the thermophilic esterase 2 from *Thermogutta terrifontis* (AF‐Est2). The table shows the analysis of noncovalent interactions and internal packing, including hydrogen bonds, hydrogen bond networks, salt bridges, and hydrophobic clusters, as well as the size and number of buried cavities.

Hydrogen bonds play a central role in protein structure by stabilizing elements of the secondary and three‐dimensional structure [75]. Analysis using CHIMERA identified 263 hydrogen bonds in Ta0887. This number is very similar to that observed in AsFAE and AF‐Est2, which have 262 and 259, respectively. When the number of hydrogen bonds is considered relative to the total number of amino acids, Ta0887 has a value of 1.12, which is slightly higher than the values observed in AsFAE and AF‐Est2, which had a normalized value of ~ 1 (Fig. 7). Therefore, it is not possible to clearly correlate the number of hydrogen bonds with the thermostability of Ta0887. The presence of hydrogen bond networks is another mechanism by which hydrogen bonds stabilize proteins by connecting distant structural elements. In this regard, Ta0887 has 15 hydrogen bond networks: 12 in the α/β‐hydrolase domain and three in the cap domain. For instance, the cap domain is stabilized by residues Glu150 and Arg154 located in α6, which form a network with Glu132 and Asn135 in α5. Two additional networks anchor the cap to the α/β‐hydrolase domain. However, the number of hydrogen bond networks present in Ta0887 is lower than that observed in AsFAE and AF‐Est2, which have 19 and 18, respectively (Fig. 7). As for salt bridges, no significant differences were observed that would suggest an impact on the thermostability of Ta0887. This is due to the presence of a relative abundance of one salt bridge per 25 residues across all three proteins (Fig. 7).

It has been proposed that hydrophobic amino acids promote protein stability by excluding water molecules from within the hydrophobic core and promoting structural compaction [83]. An important factor promoting thermostability is the presence of hydrophobic clusters consisting of 10 or more residues of Ile, Leu, or Val (branched side chains) with buried areas exceeding 500 A^2^ [84]. Ta0887 contains five hydrophobic clusters, three of which have buried areas greater than 500 A^2^ (574–3748 A^2^), which may contribute to its stability. However, no significant differences were observed when compared to AsFAE and AF‐Est2, which also have three hydrophobic clusters with buried areas greater than 500 A^2^ (Fig. 7). In contrast, when analyzing the residue content of Ile, Leu, and Val, these amino acids account for 27.2% of the total amino acid composition of Ta0887. This value is close to the 28.7% observed in AF‐Est2 and is higher than the 24.4% found in AsFAE (Fig. 7). This suggests that a higher content of hydrophobic residues, particularly those with branched side chains, promotes the thermostability of Ta0887. This is consistent with previous reports indicating that the average hydrophobicity is marginally higher in thermostable proteins [85].

Cavities are commonly found in proteins, even within the hydrophobic core [86]. The presence of internal cavities has been interpreted as packing defects with destabilizing effects [87]. Additionally, it has been proposed that a smaller number of internal cavities promotes greater stability [86]. Four internal cavities were identified in Ta0887, a number significantly lower than that observed in AsFAE and AF‐Est2, which have 14 and 13, respectively. Moreover, the total volume of the cavities in Ta0887 is 88.4 Å^3^, which is significantly lower than the 376.8 Å^3^ in AsFAE and the 288.3 Å^3^ in AF‐Est2 (Fig. 7). It has been previously noted that the presence of smaller void volumes is related to optimized packing of core regions, which in turn promotes enhanced thermal stability [88]. This suggests that the primary mechanism underlying Ta0887's thermostability is the presence of an efficiently packed hydrophobic core.

## Discussion

In this study, the Ta0887 esterase from *T. acidophilum* was successfully cloned, purified, and characterized biochemically and structurally. Based on amino acid sequence analysis with homologous sequences, the enzyme was classified into lipolytic enzyme Family V [6]. The catalytic triad of Ta0887, comprising Ser95, Asp187, and His215, features the nucleophilic Ser95 within the pentapeptide motif G‐N‐S‐Y‐G. To our knowledge, this motif is reported for the first time in characterized esterases [6]. DLS experiments revealed that Ta0887 exists as a monomer in solution, which is consistent with previous reports for AsFAE from *Alistipes shahii* (42.4% sequence similarity, PDB: 7DQ9) and AF‐Est2 from the hyperthermophilic archaeon *Archaeoglobus fulgidus* (34.5% sequence similarity, PDB: 5FRD) [35, 89]. Substrate specificity studies confirmed that Ta0887 is an esterase, as it exhibits a clear preference for short‐ to medium‐chain *p*‐NP esters, with the highest specific activity observed using *p*‐NP hexanoate (C6). The homologous esterase AF‐Est2 exhibits a similar activity profile, showing a preference for *p*‐NP valerate (C5), followed by *p*‐NP butyrate and *p*‐NP octanoate [35]. Similarly, the esterase AFEST, also identified in *A. fulgidus*, shows its highest activity against *p*‐NP hexanoate [33]. Among *p*‐NP esters, the preferred substrates for most characterized archaeal carboxylesterases are *p*‐NP valerate and *p*‐NP hexanoate [25, 26, 27, 29, 30, 32, 33]. This specificity profile is likewise consistent with that observed in lipolytic enzymes of Family V [90].

The optimal activity of Ta0887 was determined to be at 65 °C and pH 8.0, similar to that reported for the ST0071 esterase from *S. tokodaii* (70 °C, pH 8.0) [23]. Two esterases from the thermoacidophilic archaeon *S. solfataricus* P1 [27, 28] and the esterase from *S. acidocaldarius* [29] also exhibited an optimal reaction pH of 8.0. Meanwhile, AsFAE exhibits an optimal pH of 8.5 and an optimal temperature of 40 °C, consistent with the mesophilic nature of *A. shahii* [89]. The optimal reaction pH of Ta0887 (pH 8.0) differs significantly from the optimal growth pH of *T. acidophilum* (pH 1.0–2.0) and the organism's intracellular pH (pH 5.5) [44, 91]. This behavior, also observed in the esterases of *S. tokodaii* [23], *S. solfataricus* P1 [27, 28], and *S. acidocaldarius* [29], suggests that the esterases of thermoacidophilic archaea may exhibit optimal activity at pH conditions different from those of the organism. Many esters are stable under neutral or slightly alkaline conditions, so this behavior could be due to an evolutionary adaptation designed to preserve substrate stability and ensure efficient hydrolysis [23]. Furthermore, the characteristics of the Ta0887 active site may be promoting specific electrostatic interactions with the substrate or an optimal protonation state for the reaction under slightly alkaline pH conditions.

Despite their potential as a source of thermostable enzymes with industrial and biotechnological applications, few esterases from archaea have been characterized to date. In this regard, thermostability assays of Ta0887 demonstrated that it is a highly thermostable esterase, maintaining approximately 66% residual activity after 2 h of incubation at 80 °C, consistent with its *T*_m_ of 80.6 °C. It should be noted that comparable outcomes have been documented in previous studies. For instance, the ST0071 esterase maintains approximately 100% of its activity following a 2‐h incubation at 80 °C, while its activity gradually declines at higher temperatures [23]. In a similar manner, the homologous esterase AF‐Est2 from *A. fulgidus* showed a loss of activity after 30 min of incubation at temperatures above 80 °C, with complete inactivation following incubation at 95 °C [35]. Meanwhile, the Est3 esterase from *S. solfataricus* P2 retained residual activity below 20% after 2 h of incubation at 80 °C [26]. Future work will establish the potential industrial or biotechnological use of Ta0887.

Furthermore, the crystal structure of Ta0887 revealed a core domain with the canonical α/β‐hydrolase fold, typical of esterases, and an α‐helical cap domain. This cap domain is present in other esterases that share more than 31% sequence similarity with Ta0887. In these esterases, as in Ta0887, the domain is composed mainly of α‐helices, although it may also include β‐strands [35, 80, 89, 92, 93, 94]. The catalytic triad of Ta0887, located in the substrate‐binding pocket, exhibits a spatial arrangement analogous to that of AsFAE (PDB: 7DQ9), though the cavity is larger in Ta0887.

As demonstrated in previous studies, the size, shape, orientation, and spatial arrangement of the cap domain relative to the core domain are critical to determining substrate specificity. This is achieved by forming a unique substrate‐binding pocket [80, 93, 95]. This could explain Ta0887's preference for short‐ to medium‐chain substrates (C2–C8), in contrast to AsFAE, which exhibits activity only against short‐chain substrates (C2–C4) [89]. Residues in the substrate‐binding pocket also influence substrate specificity. Sayer et al. [96] demonstrated that the presence of polar and charged residues in the substrate‐binding pocket of the TtEst2 enzyme from *Thermogutta terrifontis* favors the binding of short‐chain *p*‐NP esters, such as *p*‐NP butyrate. In this regard, the composition of the active site of Ta0887 could be related to its specificity profile. On the other hand, Wei et al. [89] identified key residues in the substrate‐binding pocket of AsFAE. These residues, when mutated to alanine, significantly increased the *K*_m_, confirming the essential role of these residues in substrate recognition, binding, and affinity. In another study, the Glu154 residue of the Est19 pentapeptide motif from *Pseudomonas* sp. E2‐15 was mutated to Asp. This alteration significantly reduced the enzyme's activity toward its preferred substrate, while increasing its activity with longer‐chain substrates (*p*‐NP decanoate (C10) and *p*‐NP laurate (C12)). This substitution resulted in a deeper binding cavity in Est19, which hindered the orientation of small substrates and facilitated the access of larger substrates [8]. Furthermore, the role of specific residues of the cap domain in determining substrate specificity has been confirmed through mutations [9, 80, 89, 94]. For instance, Zhu et al. substituted the Asn121 and His125 residues in the α4 helix of the cap domain of EprEst with alanine. These mutations, which eliminated steric contacts, increased EprEst's activity toward *p*‐NP butyrate by 2.5‐ and 3.5‐fold, respectively [80]. On the other hand, Wei et al. introduced alanine mutations at Glu129 and Lys130 in the cap domain of AsFAE, demonstrating that shorter side chains improved both affinity and catalytic efficiency toward their substrates [89]. These results underscore the pivotal role of the cap domain in establishing a distinct substrate‐binding pocket that determines substrate specificity.

In conclusion, Ta0887's ability to efficiently catalyze the hydrolysis of short‐ and medium‐chain *p*‐NP esters, along with its optimal activity at 65 °C and pH 8.0 and its remarkable thermal stability, suggests a potential use for the enzyme in the food industry. This esterase could be applied in food processing, such as the modification of dairy products and the enhancement of food sensory properties through the synthesis of compounds that contribute to aroma and flavor [12]. Future evaluation of the effect of solvents, surfactants, and metal ions on Ta0887 activity, as well as the characterization of the transesterification reaction, will allow for a significant expansion of its range of biotechnological applications. Finally, the resolved crystal structure of Ta0887 provides a valuable foundation for designing point mutations in the catalytic residues, as well as in specific residues of the substrate‐binding pocket and the cap domain, in order to further explore its function and maximize its biotechnological potential.

## Conflict of interest

The authors declare no conflict of interest.

## Author contributions

AD‐R performed the experiments, analyzed and interpreted the data, performed structural analyses, and wrote the manuscript. MLL‐G performed structural analyses and revised the manuscript. GMM‐M acquired funding and revised the manuscript. LS conceived and designed the project and acquired funding. SL‐G conceived and designed the project, acquired funding, and wrote the manuscript.

## Supporting information


**Table S1.** Protein sequences used for phylogenetic analysis.
**Table S2.** Optimized Ta0887 gene for expression in *Escherichia coli*.
**Table S3.** Cleft analysis of the substrate‐binding cavity of Ta0887 compared with structural homologs identified using the DALI server.
**Figure S1.** Evolutionary conservation analysis of Ta0887 from *Thermoplasma acidophilum* based on bacterial lipolytic enzyme Family V.
**Figure S2.** Hydrophobic and electrostatic properties of Ta0887.

## Data Availability

All data supporting the results of this study are included in the article and its supplementary information. Raw experimental data will be available upon request to the corresponding author. The coordinates and structure factors of Ta0887 have been deposited in the Protein Data Bank (PDB) under the accession number 10XN.
