# Supplementary material for: Structural and biochemical insights into the thermostable esterase Ta0887 from Thermoplasma acidophilum
Source: FEBS Open Bio. 2026 Aug 21:10.1002/2211-5463.70327. Online ahead of print. doi: 10.1002/2211-5463.70327 (PMC13498779; doi:10.1002/2211-5463.70327)
Supplement: Supplementary file 1 [file FEB4-9999-0-s001.pdf]

SUPPORTING INFORMATION for

Structural and biochemical insights into the thermostable esterase Ta0887 from *Thermoplasma acidophilum*

Alejandro Delgado-Rey [a], Miriam Livier Llamas-García [a], Gabriela M. Montero-Morán [b], Leticia Santos [a] and Samuel Lara-González [a*].

[a] IPICYT, Instituto Potosino de Investigación Científica y Tecnológica A.C., División de Biología Molecular, 78216, San Luis Potosí, S.L.P., México.
[b] Universidad Autónoma de San Luis Potosí, Facultad de Ciencias Químicas, 78216, San Luis Potosí, S.L.P., México.

Corresponding author:
* Samuel Lara-González
IPICYT, Instituto Potosino de Investigación Científica y Tecnológica A.C., División de Biología Molecular, San Luis Potosí, S.L.P., México.
Tel: +52 444-834-2000 ext. 6239
E-mail address: samuel.lara@ipicyt.edu.mx

Table S1. Protein sequences used for phylogenetic analysis. The 52 protein sequences used in the phylogenetic analysis are shown along with their Uniprot accession numbers, the number of amino acids, and their sequence identity with Ta0887.

Family	Subfamily	Strain	ID Uniprot	aa	Identity (%)
I	1	*Pseudomonas aeruginosa*	P95419	311	16.51
	2	*Burkholderia glumae*	P0DUB8.1	358	16.80
	3	*Pseudomonas fluorescens SIK W1*	P26504	449	12.24
		Pseudomonas fluorescens Pf O1	Q3KCS9	562	12.24
	4	*Bacillus subtilis*	P37957	212	12.75
	5	*Bacillus stearothermophilus*	O66015	417	11.92
	6	*Staphylococcus aureus*	P10335	690	11.54
	7	*Propionibacterium acnes*	Q59644	339	14.29
	8	*Pseudoalteromonas translucida*	Q3IF07	456	12.59
		Hahella chejuensis	Q2SGZ8	411	14.84
II		*Streptomyces scabies*	P22266	345	5.80
III		*Streptomyces exfoliatus*	1JFR	262	16.67
		Streptomyces albus	Q59798	304	15.56
IV		*Pseudomonas sp B11*	O52270	308	14.71
		Moxarella sp.	P24484	433	11.72
V		*Pseudomonas oleovorans*	P26495	283	23.44
		Thermoplasma acidophilum	Q9HJS7	235	100
		Acetobacter pasteurianus	O66382	406	27.80
		Psychrobacter immobilis	Q02104	317	27.56
		Sulfolobus acidocaldarius	O73957	314	23.04
		Moraxella sp.	P24640	315	26.67

	Haemophilus influenzae	Q57427	287	22.42
VI	*Pseudomonas fluorescens*	Q53547	218	13.91
VII	*Arthrobacter oxydans*	Q01470	493	13.01
VIII	*Arthrobacter globiformis*	Q44050	375	10.38
IX	*Paucimonas lemoignei*	Q939Q9	380	10.29
X	*Thermotoga maritima*	Q9WYH1	412	12.61
XI	*uncultured bacterium*	ABY60416.1	277	13.87
XII	*uncultured bacterium*	ACB11220.1	381	7.00
XIII	*Geobacillus stearothermophilus*	AAN81912.1	176	0.00
XIV	*Caldanaerobacter subterraneus*	WP_011026365.1	414	21.18
XV	*Geobacillus thermodenitrificans*	AEN92268.1	249	18.52
XVI	*Stenotrophomonas maltophilia*	AGF29555.2	526	20.49
XVII	*Janibacter sp. R02*	ANA76126.1	423	11.76
XVIII	*Legionella spiritensis*	ABN58727.1	474	18.55
XIX	*Stenotrophomonas maltophilia*	ARJ54612.1	400	13.41
XX	*uncultured bacterium FLS18*	ACL67852.1	296	19.79
XXI	*Fervidobacterium nodosum Rt17-B1*	ABS61180.1	302	11.57
XXII	*uncultured bacterium*	ADN26553.1	397	13.74
XXIII	*uncultured bacterium*	ACJ13070.1	277	13.68
XXIV	*Mycobacterium tuberculosis H37Rv*	NP_214559.1	298	18.75
XXV	*uncultured bacterium*	ADH59407.1	330	16.96
XXVI	*uncultured organism*	AEM45109.1	397	19.53
XXVII	*uncultured organism*	AEM45123.1	448	13.82
XXVIII	*Rhodococcus erythropolis*	WP_011331609.1	467	9.52
XXIX	*uncultured bacterium*	ADZ54162	210	12.84
XXX	*uncultured microorganism*	AEH57833.1	224	13.73
XXXI	*Psychrobacter sp. MCCC 1A004444*	AGQ21328.1	329	15.27
XXXII	*uncultured bacterium*	AFR79233.1	294	15.79
XXXIII	*uncultured Acetothermia bacterium*	ANI19854.1	271	15.62
XXXIV	*Paraglaciecola chathamensis S18K6*	GAC12013.1	404	17.00
XXXV	*Caldicellulosiruptor bescii DSM 6725*	ACM59679.1	214	15.52

Table S2. Optimized Ta0887 gene for expression in *Escherichia coli*. The modified codons are shown in cyan; the codon for the S95A mutant is highlighted in yellow; the bases in red were added to incorporate the restriction sites for cloning.

Ta0887 GenBank: AL445065.1
ATG GAT AGA CAT TCA ATA ATG ACA AGT TTT GGA AAT CTT TCA TAT CTG GAA CGG CCC
GGC TCA TAT CCT CTT GTG TTT CTT CAT GGC TTA GGC GGG TCT GGA AAC AAC TGG ATC
AGA TTG GAC AGA TTT CTC GAT GGA AGA TTC AGG ATG ATC TGT TTC GAT CTG CTT GGC
CAT GGA AGA AGC GAT AAA CCA AGG GTA GAA TAT ACT GTG GAG GTT CAG GCC TCC GCA
ATC GTG GAA GCG CTT TCC AAA CTT GGA GTA AAC AGA TTC ACA CTG GTT GGA AAT TCG
TAT GGA GGA TGG ATT TCG CTA TAC ATA GCT CTT AAG AAG AAA GTT CCA GAT TAT TTA
GTG CTT GTT GAC AGC GCC GGC CTA AAT CCA ACA ATA GCA GAA CTC GGC GAT GAG AAG
CTG AAT GAA TTC GTA AAG AAG GTC ATG TCG GTA GAG GAG GGA AAC GAC GAG TAC GTC
ATC AGA AAT ATA TCC ATA AAC AAC TCA AAA GAA GAG TGG AAG ATA AAG GAT GAA GAT
CTG AGA TCA ATA AAG ACC AAA ACG CTG ATA ATC TGG GGT ACA GCA GAC AAC GTC CTC
AGC ATC GAG TAT GGG AGG AAA TTC CAC GAA CTG ATA CCG AAT AGC CTG CTT TTT GAG
ATA CCA TAT GCA AAG CAC ACG CCG CAG ATA ACG CAT CCT CAG ATC GTT GCT AGA ATA
ATA AAC GAT AAC GTC AGA ATT TGA
**Ta0887 optimized DNA sequence**
GCG GGA TCC CAT
ATG GAT CGC CAT AGC ATC ATG ACA TCG TTT GGT AAT CTG AGC TAT CTG GAA CGC CCG
GGC TCA TAT CCG CTG GTG TTT CTG CAT GGC CTG GGC GGT AGC GGT AAC AAC TGG ATT
CGC CTG GAC CGC TTT CTG GAT GGT CGC TTC CGC ATG ATC TGT TTC GAT CTG CTG GGC
CAT GGT CGC AGC GAT AAA CCG CGC GTA GAA TAT ACT GTG GAG GTT CAG GCC AGC GCA
ATC GTG GAA GCG CTG AGC AAA CTG GGT GTA AAC CGC TTC ACA CTG GTT GGT AAT AGC
TAT GGT GGT TGG ATC TCG CTG TAC ATC GCT CTG AAG AAG AAA GTT CCG GAT TAT CTG
GTG CTG GTT GAC AGC GCC GGC CTG AAT CCG ACA ATC GCA GAA CTG GGC GAT GAG AAG
CTG AAT GAG TTC GTA AAG AAG GTC ATG AGC GTA GAG GAG GGT AAC GAC GAG TAC GTC
ATC CGC AAT ATC TCC ATC AAC AAC TCA AAA GAA GAG TGG AAG ATC AAG GAT GAA GAT
CTG CGC TCA ATC AAG ACC AAA ACG CTG ATC ATC TGG GGT ACA GCA GAC AAC GTC CTG
AGC ATC GAG TAT GGT CGC AAA TTC CAC GAA CTG ATC CCG AAT AGC CTG CTG TTT GAG
ATC CCG TAT GCA AAG CAC ACG CCG CAG ATC ACG CAT CCG CAG ATC GTT GCT CGC ATC
ATC AAC GAT AAC GTC CGC ATC TAA AAG CTT GAA TTC GCG
**Ta0887 Ser95-Ala mutant**
GCG GGA TCC CAT
ATG GAT CGC CAT AGC ATC ATG ACA TCG TTT GGT AAT CTG AGC TAT CTG GAA CGC CCG
GGC TCA TAT CCG CTG GTG TTT CTG CAT GGC CTG GGC GGT AGC GGT AAC AAC TGG ATT
CGC CTG GAC CGC TTT CTG GAT GGT CGC TTC CGC ATG ATC TGT TTC GAT CTG CTG GGC
CAT GGT CGC AGC GAT AAA CCG CGC GTA GAA TAT ACT GTG GAG GTT CAG GCC AGC GCA
ATC GTG GAA GCG CTG AGC AAA CTG GGT GTA AAC CGC TTC ACA CTG GTT GGT AAT GCC
TAT GGT GGT TGG ATC TCG CTG TAC ATC GCT CTG AAG AAG AAA GTT CCG GAT TAT CTG
GTG CTG GTT GAC AGC GCC GGC CTG AAT CCG ACA ATC GCA GAA CTG GGC GAT GAG AAG
CTG AAT GAG TTC GTA AAG AAG GTC ATG AGC GTA GAG GAG GGT AAC GAC GAG TAC GTC
ATC CGC AAT ATC TCC ATC AAC AAC TCA AAA GAA GAG TGG AAG ATC AAG GAT GAA GAT
CTG CGC TCA ATC AAG ACC AAA ACG CTG ATC ATC TGG GGT ACA GCA GAC AAC GTC CTG
AGC ATC GAG TAT GGT CGC AAA TTC CAC GAA CTG ATC CCG AAT AGC CTG CTG TTT GAG
ATC CCG TAT GCA AAG CAC ACG CCG CAG ATC ACG CAT CCG CAG ATC GTT GCT CGC ATC
ATC AAC GAT AAC GTC CGC ATC TAA AAG CTT GAA TTC GCG

Table S3. Cleft analysis of the substrate-binding cavity of Ta0887 compared with structural homologs identified using the DALI server.

Protein	PDB	Length (residues)	Z score[a]	RMSD[a] (Å)	Substrate-binding cavity volume (Å^3)[b]
Ta0887	10XN	235	-	-	3293.2
EaEst	5H3H	272	29.5	2.1	966.1
TtEst	4UHC	282	28.2	2.3	1480.4
AsFAE	7DQ9	265	27.2	2.3	2348.2
Ade1	8V16	274	27.3	2.4	5423.6
AF-Est2	5FRD	260	26.8	2.2	2249

[a]The values were obtained from the analysis using the DALI server.

[b]The volume of the substrate-binding cavity was estimated using PDBsum tools.

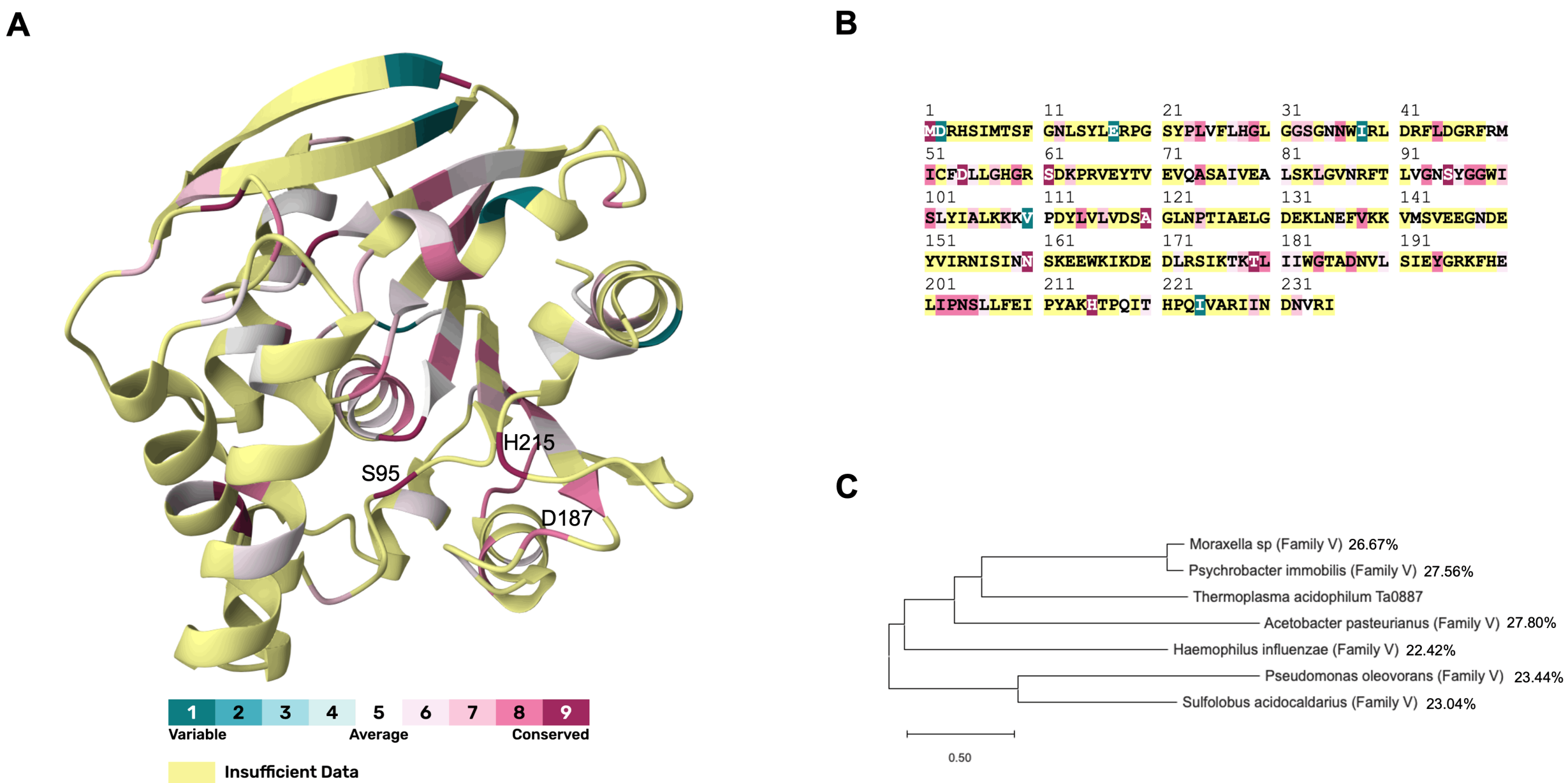

Figure S1. Evolutionary conservation analysis of Ta0887 from *Thermoplasma acidophilum* based on bacterial lipolytic enzyme Family V. **(A)** Three-dimensional structure of Ta0887 colored according to residue-specific evolutionary conservation scores calculated with ConSurf using the Bayesian inference method. Conservation scores were estimated from a multiple sequence alignment comprising representative members of bacterial lipolytic enzyme Family V. Residues are colored according to the ConSurf conservation scale, ranging from variable (turquoise, grades 1–4) to highly conserved (magenta, grades 6–9), whereas residues shown in yellow indicate positions with insufficient phylogenetic information for reliable conservation estimation. The catalytic triad residues Ser95, Asp187, and His215 are indicated. **(B)** Multiple sequence alignment of Ta0887 and representative members of bacterial lipolytic enzyme Family V generated with MAFFT and used as the input for ConSurf analysis. Amino acid residues are colored according to the Bayesian conservation scores calculated by ConSurf, highlighting the conservation pattern across the family, including the catalytic GXSXG motif and surrounding residues. **(C)** Maximum likelihood phylogenetic tree of Ta0887 and representative members of bacterial lipolytic enzyme Family V constructed in MEGA12 from a MUSCLE multiple sequence alignment using the Le and Gascuel amino acid substitution model. Pairwise sequence identity values relative to Ta0887, calculated using Clustal Omega, are indicated for each representative enzyme.

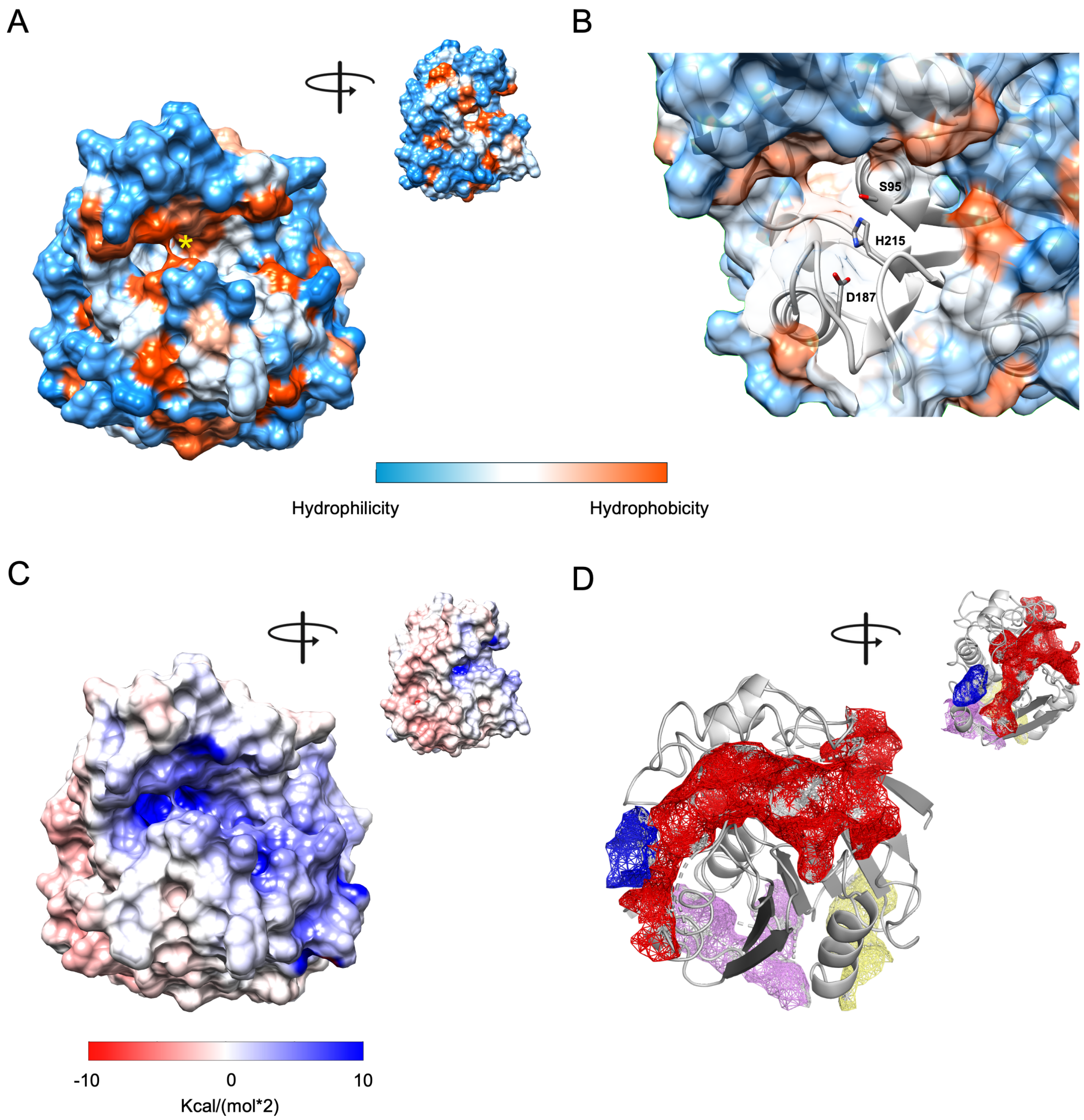

Figure S2. Hydrophobic and electrostatic properties of Ta0887. **(A)** Hydrophobic surface representation of Ta0887. The hydrophobic region (orange-red) surrounding Ser95 (yellow asterisk) is visible at the center of the substrate-binding pocket; the hydrophilic regions are shown in dodger blue. **(B)** A close-up view of the hydrophobic region previously mentioned in (A). The residues of the catalytic triad are represented as sticks. Ser95 is located at the bottom of the cavity within a hydrophobic pocket. The figure in (A) was rotated 45° to the left along the vertical axis, and a cross-section of the protein is shown. **(C)** Surface representation of Ta0887 showing its calculated electrostatic potential. Regions with a positive charge are shown in blue and regions with a negative charge in red. The substrate-binding cavity is predominantly positively charged. **(D)** The substrate-binding surface of Ta0887 is shown, with a volume of 3293.2 Å^3 as determined by cleft analysis using PDBsum tools. Panels (A), (B), and (C) were generated with USFC CHIMERA; panel (D) was generated with Pymol.
